# A Persistent Flexural Dermatosis Unmasking Adult Langerhans Cell Histiocytosis: A Case Report

**DOI:** 10.7759/cureus.113802

**Published:** 2026-08-02

**Authors:** Lamis El Yamani, Ouissal Hormi, Salma Moujahid, Khadija Imam, Nassiba Zerrouki, Nada Zizi

**Affiliations:** 1 Dermatology, Mohammed VI University Hospital of Oujda, Oujda, MAR; 2 Dermatology, Allergology and Venerology, Mohammed VI University Hospital of Oujda, Oujda, MAR

**Keywords:** cutaneous langerhans cell histiocytosis, erosive dermatosis, intertriginous lesions, langerhans cell histiocytosis, vulvar involvement

## Abstract

Langerhans cell histiocytosis (LCH) is a rare histiocytic neoplasm that may present with isolated cutaneous involvement or multisystem disease. In adults, cutaneous manifestations are often polymorphic and may mimic more common dermatologic conditions, leading to delayed diagnosis. A 41-year-old woman with a history of treated breast carcinoma presented with a two-year history of chronic erosive lesions involving the vulvar and intertriginous regions. Additional lesions affected the scalp, retroauricular folds, umbilicus, and external auditory canals. Histopathological examination of a vulvar biopsy, supported by positive CD1a and S100 immunostaining, confirmed the diagnosis of LCH. A comprehensive extension workup revealed no evidence of visceral, pulmonary, lymph node, or skeletal involvement. The patient was treated with topical clobetasol propionate, local wound care, and treatment of secondary bacterial superinfection, resulting in marked improvement of the vulvar and intertriginous lesions during follow-up. Adult cutaneous LCH remains a diagnostic challenge because of its ability to mimic inflammatory and infectious dermatoses, particularly when flexural and genital areas are affected. Histopathological examination with immunohistochemical confirmation is essential for establishing the diagnosis and guiding further evaluation. LCH should be considered in the differential diagnosis of persistent erosive vulvar and intertriginous lesions that are unresponsive to conventional therapies. Early biopsy is crucial to avoid diagnostic delay and to ensure appropriate staging and management.

## Introduction

Langerhans cell histiocytosis (LCH) is a rare clonal disorder of dendritic cells characterized by the accumulation of CD1a-, langerin (CD207)-, and S100-positive histiocytes within affected tissues [1]. The disease is exceptionally uncommon in adults, with an estimated incidence of only 1-2 new cases per million people each year [2]. Molecular studies have demonstrated recurrent activation of the MAPK signaling pathway, supporting its current classification as an inflammatory myeloid neoplasm [1-3]. Adult LCH encompasses a broad clinical spectrum ranging from single-organ disease to multisystem involvement [2,4]. Distinguishing between single-system and multisystem disease is essential, as it guides staging investigations, therapeutic management, and prognosis. Although cutaneous involvement may occur in both single-system and multisystem disease, isolated vulvar and intertriginous involvement in adults is exceptionally rare. The diversity and nonspecific nature of its manifestations, particularly when confined to the skin, often contribute to substantial diagnostic delay. Cutaneous lesions, especially in seborrheic or intertriginous areas, may mimic a wide range of more common disorders, including inflammatory dermatoses (such as inverse psoriasis and Hailey-Hailey disease), infectious conditions (including candidal or bacterial intertrigo and erythrasma), and other inflammatory blistering diseases such as pemphigus vegetans, making early recognition particularly challenging. We report the case of an adult woman with cutaneous and mucosal LCH presenting as chronic erosive vulvar and intertriginous lesions in the setting of previous breast carcinoma.

## Case presentation

A 41-year-old Moroccan woman was admitted to our dermatology department with a two-year history of persistent erosive lesions affecting the vulvar and intertriginous areas. She had a previous history of right breast carcinoma diagnosed in December 2021, which had been treated with breast-conserving surgery, ipsilateral axillary lymph node dissection, adjuvant chemotherapy, and radiotherapy, all completed before the onset of the cutaneous lesions. She had a normal body mass index, was a non-smoker, and had no history of immunodeficiency or ongoing immunosuppressive treatment. At presentation, she was receiving adjuvant tamoxifen therapy.

**Figure 1:**
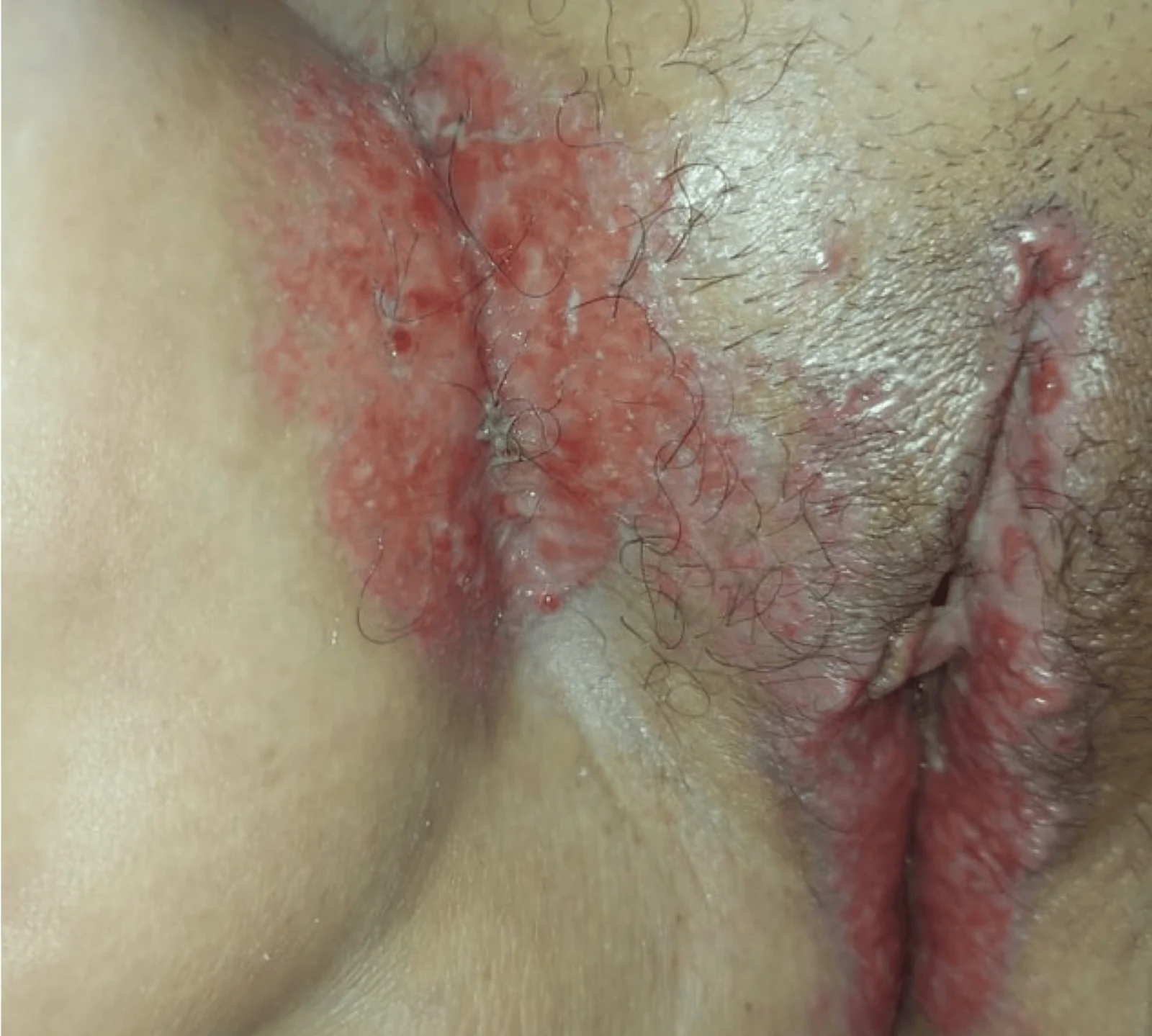
Inguinal involvement in cutaneous Langerhans cell histiocytosis. Slightly infiltrated erythematous plaques with superficial erosions involving the bilateral inguinal folds.

**Figure 2:**
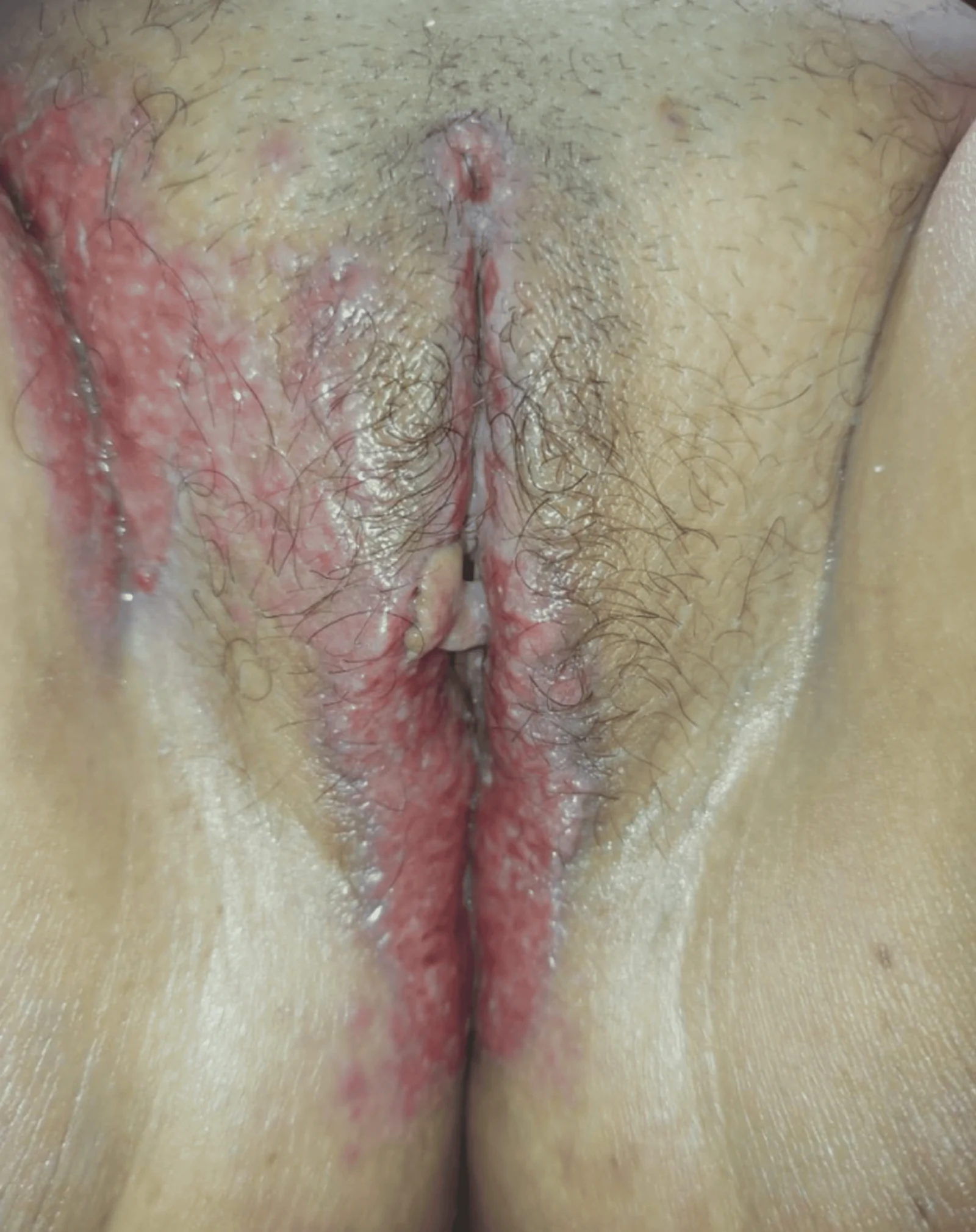
Clinical presentation of genital Langerhans cell histiocytosis. Erythematous erosions with a superficial fibrinous coating involving the vulva.

The eruption initially consisted of pruritic erythematous papules located in the inguinal folds, which gradually enlarged and became erosive. The lesions remained continuously present over the following two years, with progressive enlargement and extension to the vulvar, intergluteal, umbilical, retroauricular, external auditory canal, and scalp regions, without episodes of partial or complete spontaneous remission. The patient initially consulted a general practitioner and subsequently a gynecologist. Based on the clinical presentation, the lesions were considered most consistent with a chronic infectious dermatosis, and she was successively treated with topical ketoconazole 2% cream for four weeks, oral fluconazole 150 mg once weekly for four weeks, and oral amoxicillin-clavulanic acid (3 g/day) for 10 days, without significant clinical improvement. The persistence of the lesions despite these treatments, together with their progressive extension to multiple anatomical sites, prompted referral to our department. At presentation, the patient reported persistent pruritus but denied pain, burning sensation, vaginal discharge, or malodor. On examination, there were extensive erythematous and eroded plaques involving both inguinal folds, measuring approximately 8 × 5 cm on each side, the intergluteal cleft, and the vulvar area, where a large erosive plaque measuring approximately 10 × 4 cm involved the labia majora, labia minora, and perineum (Figures 1, 2). Similar infiltrated erosive plaques with overlying crusts were observed in the umbilical and retroauricular regions. The external auditory canals showed multiple erythematous infiltrated papules with fine scaling. The scalp exhibited diffuse erythematous infiltrated plaques covered by greasy scales and crusts, clinically resembling seborrheic dermatitis. The oral mucosa was spared, and there was no palpable lymphadenopathy or hepatosplenomegaly.

**Figure 3:**
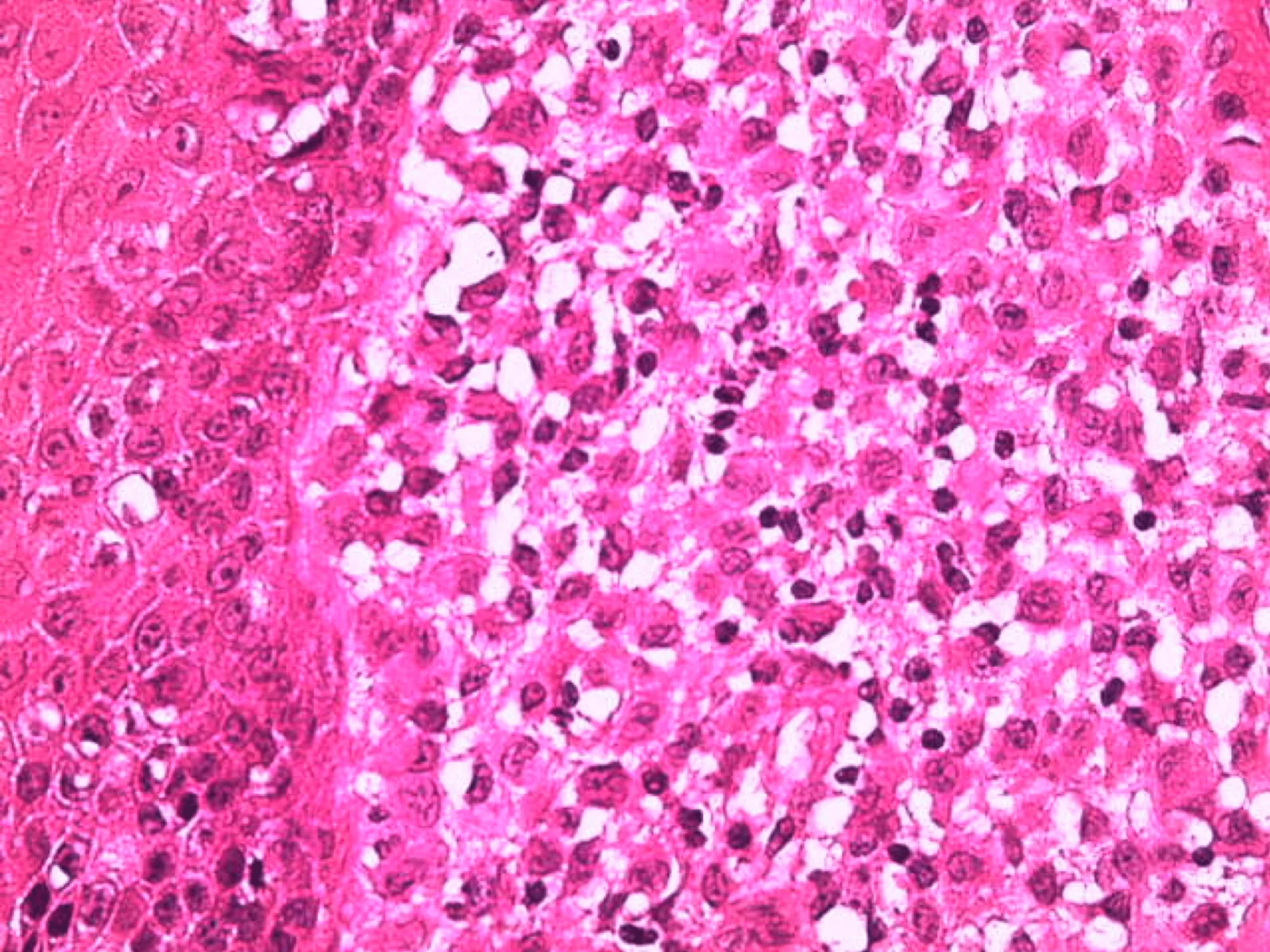
Histopathological features of cutaneous Langerhans cell histiocytosis. A dense dermal infiltrate composed of large histiocytic cells with abundant pale cytoplasm and characteristic grooved nuclei, admixed with inflammatory cells. These histopathological findings are consistent with Langerhans cell histiocytosis (hematoxylin and eosin, ×400).

**Figure 4:**
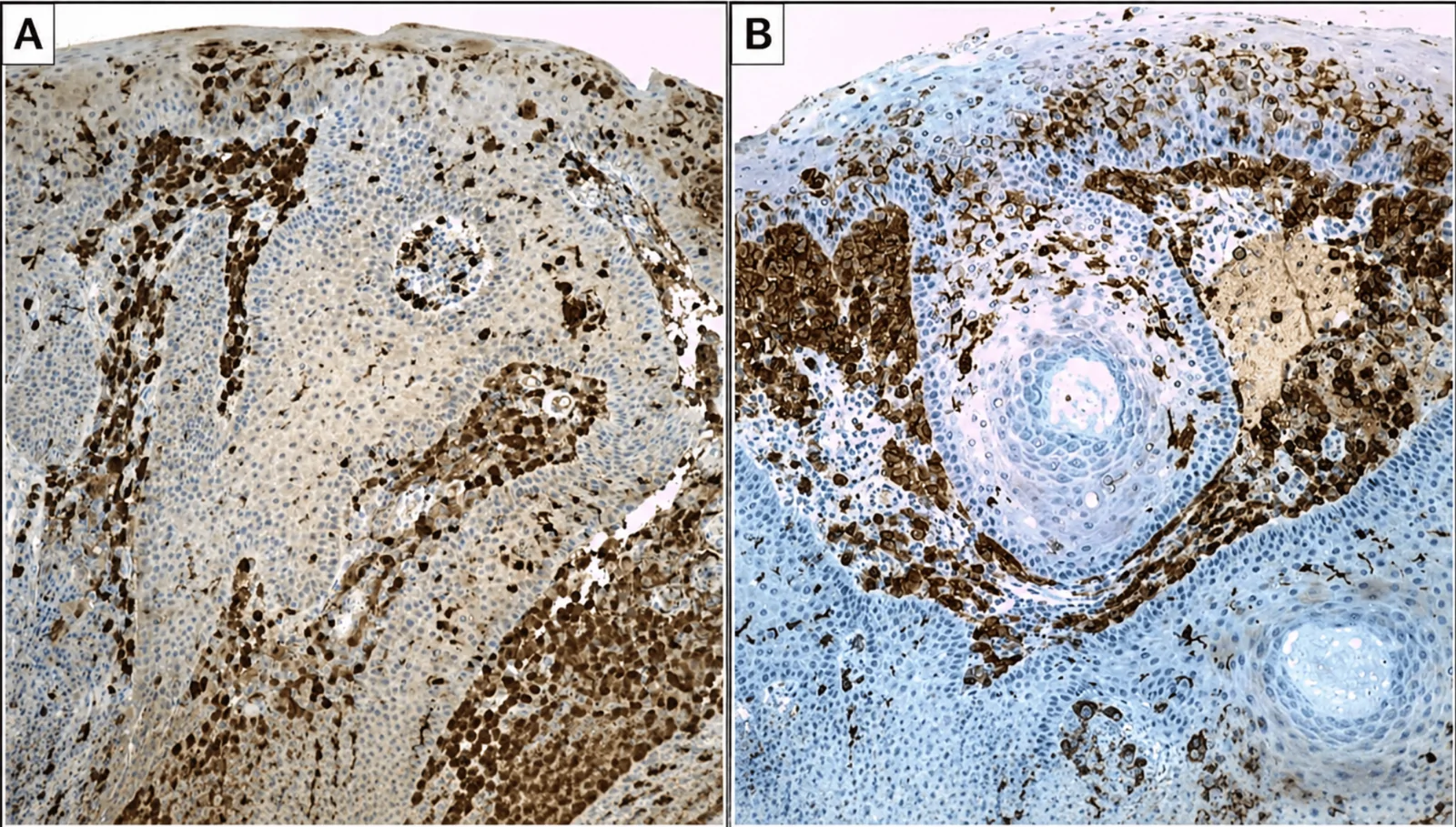
Immunohistochemical confirmation of Langerhans cell histiocytosis. Immunohistochemical staining of the skin lesion demonstrating the characteristic immunophenotype of Langerhans cell histiocytosis. (A) Strong and diffuse positivity for S100 protein within the lesional histiocytic cells. (B) Diffuse membranous and cytoplasmic expression of CD1a by the infiltrating cells, confirming their Langerhans cell lineage. The pale rounded structures visible in the background correspond to normal hair follicles and are not part of the diagnostic histopathological findings (original magnification ×100).

Given the chronic, multifocal, and treatment-refractory nature of the disease, a vulvar biopsy was subsequently performed. Histopathological examination revealed a dermal infiltrate composed of atypical histiocytic cells (Figure 3). Immunohistochemical analysis demonstrated strong positivity for CD1a, S100, and Langerin (CD207), confirming the diagnosis of LCH (Figure 4). Molecular analysis for the *BRAF V600E* mutation was not performed, precluding further characterization of the molecular profile and assessment of potential eligibility for targeted therapy.

After diagnostic confirmation, a staging assessment was performed. Complete blood count was normal, with preserved leukocyte, hemoglobin, and platelet levels. Renal and hepatic tests, as well as inflammatory markers, were within normal ranges. Serologic tests for hepatitis B, hepatitis C, and syphilis were negative.

Thoraco-abdomino-pelvic computed tomography showed no pulmonary nodules, hepatic or splenic lesions, significant lymphadenopathy, or other visceral findings suggestive of systemic LCH. Skeletal evaluation, including radiographs of the humeri, femora, spine, ribs, and pelvis, did not reveal osteolytic lesions.

The overall evaluation showed no evidence of pulmonary, visceral, lymph node, hematologic, or skeletal involvement (Table 1). The diagnosis of adult-onset cutaneous and mucosal LCH was therefore retained. Treatment consisted of topical clobetasol propionate combined with local wound care. At the one-year follow-up, the patient achieved more than 95% clinical improvement, with marked reduction in lesion size, complete resolution of the erosive lesions, and complete resolution of pruritus. No new cutaneous, mucosal, or systemic manifestations were observed during follow-up.

**Table 1:** Staging workup and results following the diagnosis of adult cutaneous and mucosal Langerhans cell histiocytosis. Summary of laboratory investigations, serologic tests, and imaging studies performed to assess extracutaneous involvement. No evidence of pulmonary, visceral, lymph node, hematologic, or skeletal disease was identified.

Assessment	Findings
Laboratory tests	Normal complete blood count, renal and liver function tests, and inflammatory markers
Serologies	Negative for hepatitis B, hepatitis C, and syphilis
Thoraco-abdomino-pelvic CT	No pulmonary, visceral, or lymph node involvement
Skeletal survey	No osteolytic lesions
Overall staging	No evidence of multisystem involvement (pulmonary, visceral, hematologic, lymph node, or skeletal disease)

## Discussion

LCH is a rare clonal disorder of myeloid dendritic cells characterized by the accumulation of pathologic cells sharing the immunophenotypic features of epidermal Langerhans cells. Although LCH predominantly affects children, adult cases are increasingly recognized and likely remain underdiagnosed because of their heterogeneous clinical presentation [1,2].

The clinical spectrum of LCH ranges from single-system disease to multisystem involvement affecting the skeleton, lungs, liver, spleen, lymph nodes, and central nervous system [2,3]. Cutaneous manifestations represent one of the most common presentations in adults and may occur either as an isolated form or in association with systemic disease [4].

The diagnosis of cutaneous LCH is often challenging because of its diverse clinical manifestations. Skin lesions frequently involve seborrheic and flexural regions, including the scalp, retroauricular folds, axillae, groins, and anogenital area, where they may mimic more common inflammatory dermatoses such as seborrheic dermatitis, psoriasis, chronic candidiasis, Hailey-Hailey disease, or pemphigus vegetans [4-6]. In the present case, the persistence of erosive vulvar and inguinal lesions despite multiple treatments contributed to a diagnostic delay of nearly two years, highlighting the importance of maintaining a high index of suspicion in refractory intertriginous dermatoses.

Genital involvement is an uncommon manifestation of adult LCH and has been reported mainly in isolated case reports and small case series [7,8]. Because vulvar lesions may resemble infectious, inflammatory, or neoplastic disorders, diagnosis is frequently delayed. In our patient, the concomitant involvement of the vulva, scalp, retroauricular folds, umbilicus, and external auditory canals suggested a unifying systemic dermatologic process and prompted further investigation.

In our patient, the main differential diagnoses included inverse psoriasis, chronic candidal or bacterial intertrigo, erythrasma, Hailey-Hailey disease, and pemphigus vegetans. The lack of response to standard treatments, together with the presence of lesions at other sites such as the scalp and retroauricular folds, led to biopsy and immunohistochemical analysis, which confirmed LCH.

Histopathological examination remains the cornerstone of diagnosis. Typical findings include an infiltrate of histiocytic cells with irregular or grooved nuclei accompanied by variable numbers of eosinophils. Immunohistochemical demonstration of CD1a, langerin (CD207), and S100 expression confirms the diagnosis [2,3,9]. In our case, the characteristic histological features together with diffuse CD1a and S100 positivity established the diagnosis of LCH.

Because cutaneous lesions may represent the first manifestation of systemic disease, a thorough staging assessment is recommended in all newly diagnosed adult patients to distinguish single-system from multisystem disease, guide therapeutic decision-making, and establish prognosis [2,10]. Bone and pulmonary involvement are the most common extracutaneous manifestations in adulthood [10,11], making their systematic evaluation particularly important even in patients presenting with apparently isolated skin lesions. Our patient underwent comprehensive staging, including thoraco-abdomino-pelvic imaging and skeletal evaluation, which showed no evidence of pulmonary, lymphatic, visceral, or osseous involvement. The absence of extracutaneous disease allowed classification as single-system cutaneous and mucosal LCH, an uncommon presentation in adults that is generally associated with a more favorable prognosis and may be managed with skin-directed therapy.

An additional noteworthy feature of this case is the patient’s history of breast carcinoma. The coexistence of LCH and malignant neoplasms has been reported in several studies, although the nature of this association remains poorly understood [12,13]. Proposed mechanisms include treatment-related immune dysregulation, shared molecular pathways, or coincidental occurrence. Recent molecular studies have demonstrated recurrent activation of the MAPK pathway, particularly involving *BRAF* mutations, supporting the neoplastic nature of LCH; however, whether these alterations contribute to the observed association with solid malignancies remains unclear [9,14]. In our patient, no evidence of recurrent breast cancer was identified, and the cutaneous lesions were unequivocally attributable to LCH.

Due to the rarity of adult cutaneous LCH, therapeutic recommendations are largely based on retrospective studies and expert consensus. Reported treatment options include topical or systemic corticosteroids, methotrexate, thalidomide, phototherapy, and targeted therapies directed against the MAPK pathway in selected patients [2,10,14]. In the present case, topical corticosteroid therapy combined with local supportive care resulted in marked clinical improvement, supporting the use of conservative treatment in localized disease.

This case emphasizes the broad clinical spectrum of adult cutaneous LCH and illustrates the diagnostic difficulties posed by chronic erosive lesions of the vulvar and intertriginous regions. Early biopsy with appropriate immunohistochemical analysis is essential to establish the diagnosis, perform adequate staging, and avoid prolonged diagnostic delay.

Limitations

This report has several limitations. As a single-case observation, its findings cannot be generalized to the broader population of patients with cutaneous LCH. In addition, molecular analysis for *BRAF* mutations was not performed, precluding further characterization of the underlying molecular profile and potential therapeutic implications. Finally, although the patient showed sustained clinical improvement, the follow-up duration remains relatively limited and does not allow assessment of long-term outcomes, including the risk of recurrence or progression to systemic disease.

## Conclusions

This case underscores the diagnostic complexity of adult-onset cutaneous and mucosal LCH, particularly when it presents as chronic erosive lesions involving flexural and genital areas. Because its clinical features may closely resemble those of more common inflammatory or infectious dermatoses, recognition of the disease is often delayed. In patients with persistent intertriginous or vulvar lesions that fail to improve with standard treatments, histological examination should be considered early in the diagnostic workup. Prompt diagnosis not only allows appropriate therapeutic management but also facilitates the assessment of possible extracutaneous involvement and long-term surveillance. In addition, the coexistence of LCH with solid malignancies, as observed in our patient with a history of breast carcinoma, highlights the need for continued clinical vigilance, although the nature of this association remains uncertain.
